# Randomized Controlled Trial of a Community Health Worker Self-Management Support Intervention Among Low-Income Adults With Diabetes, Seattle, Washington, 2010–2014

**DOI:** 10.5888/pcd14.160344

**Published:** 2017-02-09

**Authors:** Karin Nelson, Leslie Taylor, Julie Silverman, Meghan Kiefer, Paul Hebert, Dan Lessler, James Krieger

**Affiliations:** 1 VA Health Services Research and Development, Seattle-Denver Center of Innovation for Veteran-Centered and Value-Driven Care, Seattle, Washington; 2 VA Puget Sound Health Care System, General Internal Medicine Service, Seattle, Washington; 3 University of Washington, School of Medicine, Seattle, Washington; 4 University of Washington, School of Public Health, Seattle, Washington; 5 Public Health — Seattle and King County, Seattle, Washington

## Abstract

**Introduction:**

Community health workers (CHWs) can improve diabetes outcomes; however, questions remain about translating research findings into practical low-intensity models for safety-net providers. We tested the effectiveness of a home-based low-intensity CHW intervention for improving health outcomes among low-income adults with diabetes.

**Methods:**

Low-income patients with glycated hemoglobin A_1c_ (HbA_1c_) of 8.0% or higher in the 12 months before enrollment from 3 safety-net providers were randomized to a 12-month CHW-delivered diabetes self-management intervention or usual care. CHWs were based at a local health department. The primary outcome was change in HbA_1c_ from baseline enrollment to 12 months; secondary outcomes included blood pressure and lipid levels, quality of life, and health care use.

**Results:**

The change in HbA_1c_ in the intervention group (n = 145) (unadjusted mean of 9.09% to 8.58%, change of −0.51) compared with the control group (n = 142) (9.04% to 8.71%, change of −0.33) was not significant (*P* = .54). In an analysis of participants with poor glycemic control (HbA_1c_ >10%), the intervention group had a 1.23-point greater decrease in HbA_1c_ compared with controls (*P* = .046). For the entire study population, we found a decrease in reported physician visits (*P* < .001) and no improvement in health-related quality of life (*P* = .07) in the intervention group compared with the control group.

**Conclusion:**

A low-intensity CHW-delivered intervention to support diabetes self-management did not significantly improve HbA_1c_ relative to usual care. Among the subgroup of participants with poor glycemic control (HbA_1c_ >10% at baseline), the intervention was effective.

## Introduction

Diabetes is epidemic, and low-income and racial/ethnic minority populations have a high disease burden (1). Translating effective diabetes self-management interventions into diverse settings is a public health challenge (2). Using community health workers (CHWs) may be an effective approach with disadvantaged populations (3,4). A recent meta-analysis reported modest effects for CHW interventions for patients with diabetes (3). Many previous studies were conducted in single sites or targeted racial or ethnic minority groups. Studies varied widely in the intensity of the CHW intervention, with visit frequencies ranging from 4 to 36 (3). Learning whether CHW interventions can be implemented in community settings with multiple health care providers using a low-intensity (and more affordable) intervention with similar results is the next step for determining the role of CHWs in diabetes management.

Multiple models exist for organizing the delivery of CHW services in communities with complex, multisite health care delivery systems. One approach is for each delivery system to have its own CHWs. Another is for a central organization, such as a local health department, to offer CHW services to multiple health care entities, which may offer opportunities for greater efficiency and coordination, especially for smaller health systems and clinics. Health departments may also have superior knowledge of community-based resources and how to connect to those resources. Therefore, we developed and evaluated the Peer Support for Achieving Independence in Diabetes (Peer-AID) project to determine the effectiveness of a low-intensity CHW diabetes self-management intervention in which a local health department provided CHW services to a community health center, public hospital, and US Department of Veterans Affairs (VA) hospital (5).

## Methods

### Trial design

A description of the study design and intervention components were previously published (5). Peer-AID developed a model in which the local health department (Public Health — Seattle King County [PHSKC]) provided CHW services to a diverse set of clinics. PHSKC worked with clinical sites to identify potential clients and coordinated care by 1) alerting providers via telephone or fax to clinical issues that arose either in the CHW visits or in the team meetings held every other week and 2) encouraging clients to follow up with their clinic care providers. Out-of-range values for glycated hemoglobin A_1c_ (HbA_1c_), blood pressure, or depression screening were either faxed or sent via encrypted email to clinic care providers. The CHWs were full-time employees recruited from the communities that the project served, were native Spanish speakers, and had high school or equivalent degrees. The CHWs received 40 hours of classroom sessions, including training to use an automated blood pressure monitor. A health educator and Certified Diabetes Educator (CDE) provided clinical support in bi-weekly meetings, and a manager provided supervision and operations oversight.

### Participants

Participants were recruited from Harborview Medical Center (HMC), the VA Puget Sound Health Care System (VAPSHCS), and Sea Mar Community Health Centers. Harborview Medical Center is the largest public hospital safety-net provider in King County. VAPSHCS is a tertiary referral hospital serving veterans from a multistate area. Sea Mar Community Health Centers are community-based clinics specializing in service to Latinos. The University of Washington and the VA Puget Sound institutional review boards approved the study. Written informed consent was obtained from all participants.

The electronic health records from each clinic site were queried to identify potentially eligible participants, defined as having a diagnosis of type 2 diabetes and whose most recent HbA_1c_ was 8.0% or greater (performed in the 12 months before enrollment), having a household income of less than 250% of the federal poverty level, being aged 30 to 70 years, being English-speaking or Spanish-speaking, and residing in King County, Washington. Exclusion criteria included current participation in another diabetes study; completing diabetes education in the prior 3 years; being homeless or planning to move out of the area; having a serious illness, including cancer, end-stage renal disease requiring hemodialysis, or dementia; or being unable to give informed consent.

After obtaining informed consent and collecting baseline data, we randomly assigned participants to intervention or usual care control arms in a 1:1 ratio using a stratified, permuted block design with varying block size. Stratification was by clinic site. The design of the intervention made it impossible to blind participants and staff to group assignment.

### Intervention

Baseline data collection was performed and informed consent was obtained in participants’ homes. Baseline assessments were completed between September 2010 and May 2013, and 1-year exit visits were completed in November 2014.

The intervention took place in participants’ homes and comprised 4 mandatory home visits that took place 0.5, 1.5, 3.5, and 7 months after the enrollment visit with an optional visit at 10 months. At each visit, the CHW assessed diabetes self-management using a structured interview (5). CHWs worked with clients to set health goals (6,7) and develop an action plan for diabetes self-management activities. At each visit, the CHWs completed an encounter form, which documented the health goal that was the focus of the visit and the self-management strategies that were discussed. The encounter forms were reviewed by the project CDE. Monthly audits were completed to ensure that each participant received the required components of the intervention.

### Outcome and other measures

The primary outcome of the study was HbA_1c_ values. We obtained a baseline HbA_1c_ measurement and lipid panel at the first home visit using a mail-in finger-stick kit (CheckUp America Diabetes and Cholesterol Test Panel [Home Access Health Corporation]), which allows an individual to self-test with a finger-stick sample of blood and includes a lipid panel and HbA_1c_ test. All testing is performed in a laboratory regulated by Clinical Laboratory Improvement Amendments and certified by the College of American Pathologists. This method is accurate and reliable, similar to venous blood sampling done by a professional laboratory (8). We collected baseline home-testing data for 259 of 287 participants (90%) and exit home-testing on 261 of 287 participants (91%). Clinic values were used when home-testing kit values were not available (n = 28 at baseline, n = 24 at exit). We initially attempted to collect fasting samples, but because of logistical challenges (eg, participant forgot to fast), most samples were nonfasting. We used the ratio of total cholesterol to HDL cholesterol to measure lipid control (9).

Blood pressure and weight were measured by the CHW at baseline and exit. Body weight was measured in light street clothes without shoes on calibrated electronic scales that measured up to 300 pounds. All participants over 300 pounds had a self-reported weight (n = 21). Standing height was obtained at baseline, to allow for calculation of body mass index (BMI). Blood pressure was measured 3 times during both the baseline and exit visit by using an automated calibrated blood pressure cuff (Omron Automatic Blood Pressure Monitor 5 Series) with the participant in a seated position after 5 minutes of rest. The average of the 3 blood pressure readings were used as the baseline and exit values.

Secondary outcomes included health-related quality of life (HRQOL) measured with the SF-12 Physical Component Summary (PCS) and Mental Component Summary (MCS) scales (10) and the social burden subscale of the Diabetes-39 instrument to measure diabetes-specific HRQOL (11). Health care use was documented by self-reported number of outpatient clinic visits, hospitalizations, and emergency department encounters in the prior year. Level of physical activity was assessed by using the International Physical Activity Questionnaire to classify participants as having high (eg, >60 minutes of moderate-intensity activity per day), moderate (eg, half-hour of at least moderate-intensity physical activity on most days), or low levels (12). Medication adherence was assessed by using a standardized participant interview (13). We included a measure of depressive symptoms (the Patient Health Questionnaire depression scale [PHQ-8]) (14), because of the strong link between depression and poor self-care among people with diabetes (15).

We collected data on potential covariates associated with diabetes control and self-care. Demographic covariates included self-reported age, sex, marital status, education, and race/ethnicity. All participants were asked a screening question on income, allowing classification as being below the 250% threshold for the federal poverty level. We asked participants to describe their race as white, black, American Indian or Alaskan Native, Asian, multiracial, or other and their ethnicity as Hispanic or non-Hispanic.

### Statistical methods

The sample size was based on detecting a difference in change in HbA_1c_ of 0.7% between groups with 80% power and a 2-sided α of .05, allowing for a dropout rate of 15%.

Unadjusted comparisons between intervention groups were calculated using *t* tests for continuous outcomes and χ^2^ tests for categorical outcomes. A linear model was used for the primary intention-to-treat analysis that compared HbA_1c_ changes from baseline to 12 months by intervention group, controlling for baseline HbA_1c_, clinic site, and BMI (which was unbalanced at baseline). To determine whether the intervention effect depended on baseline HbA_1c_ levels, we examined the interaction between the treatment indicator and baseline HbA_1c_ level. In prespecified subgroup analyses for those with very poorly controlled diabetes at baseline, we analyzed 2 groups: those with an HbA_1c_ level higher than 9% and those with an HbA_1c_ level higher than 10%). An HbA_1c_ level higher than 9% is a commonly used definition of poor control, as reflected in national guidelines, performance measures, and national treatment goals (16–18). A subgroup of participants with HbA_1c_ level higher than 10% was examined because of the evidence showing that cost savings are achieved in this subgroup only among cohorts of patient with improved diabetes control (19). HbA_1c_ levels higher than 10% is a marker of severe hyperglycemia, for which insulin is recommended (20). Secondary analyses used linear, logistic, and zero-inflated Poisson models for continuous, binary, and count data outcomes, respectively. Although analyses on primary and secondary outcomes used complete-case methods, we ran additional analyses on secondary outcomes using multiple imputation chained equations methods (19) because of missing data. Because there were no differences in the results, we tabulated only the complete-case analyses.

## Results

### Participant flow and baseline data

**Figure 1:**
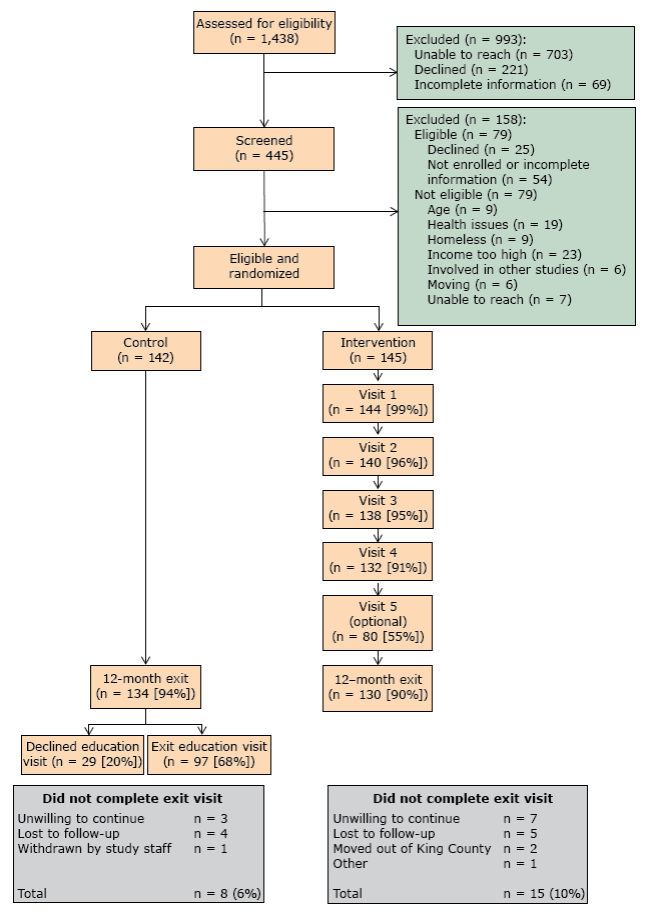
Recruitment of patients for Peer Support for Achieving Independence in Diabetes (Peer-AID) trial using community health workers to provide self-management support among low-income adults with diabetes, Seattle, Washington, 2010–2014.

Of the 1,438 patients identified as potentially eligible, 49% could not be contacted (n = 703), 15% declined participation (n = 221), and 5% had incomplete screening information (n = 69) (Figure 1). From the remaining 445, we excluded those who were eligible but declined to participate or for whom information was incomplete, and we excluded those who did not meet the eligibility criteria. Of the 287 randomized participants, 262 completed the 12 months follow up (91% completion rate).

We met our recruitment target of 287 individuals. Adherence to the intervention protocol was high: 86% of all visits were completed within the period specified and only 1.2% of visits were incomplete. No adverse events occurred.

### Baseline demographics and clinical characteristics

Intervention and control groups were similar at baseline on sociodemographic and most clinical characteristics (Table 1). Participants had low income and were from diverse racial and ethnic backgrounds. The mean BMI for the intervention group was 32.5 (vs 34.7 in the control group, *P* = .04). The proportion of individuals with self-reported fair or poor health was higher in the control group. The mean duration of diabetes was more than 10 years, and the population had high rates of depression and comorbid diseases. Many participants reported low levels of medication adherence and physical activity.

**Table 1:** Baseline Population Characteristics of Low-Income Participants With Poorly Controlled Diabetes, Peer Support for Achieving Independence in Diabetes (Peer-AID), Seattle, Washington, 2010–2014

Characteristics	Control (n = 142)	Intervention (n = 145)	Total Population (n = 287)	*P* Value^a^
**Demographic Characteristics**				
**Female, %**	52.8	44.8	48.8	.18
**Age, mean (SD), y**	51.7 (9.5)	53.3 (9.1)	52.5 (9.3)	.13
**Less than high school education, %**	32.6	35.9	34.3	.56
**Married, %**	32.4	33.1	32.8	.90
**Employed, %**	25.4	31.7	28.6	.23
**Hispanic, %**	40.9	44.8	42.9	.50
**Race, % **				
White	42.8	48.3	45.6	.61
Black	29.0	24.1	26.5	
American Indian or Alaska Native	7.3	4.8	6.0	
Asian	5.8	5.5	5.7	
Multiracial	5.0	9.0	7.0	
Other	10.1	8.3	9.2	
**Clinical Characteristics**				
**BMI, kg/m^2^, mean (SD)**	34.7 (9.4)	32.5 (8.3)	33.6 (8.9)	.04
**Obese, %^b^**	63	54	59	.10
**Use oral agents for diabetes, %**	100.0	98.3	99.1	.16
**Use insulin, %**	63	58	61	.39
**Duration of diabetes, mean (SD), y**	11.4 (8.2)	10.6 (6.9)	10.9 (7.6)	.38
**Low medication adherence, %**	44.4	45.1	44.7	.91
**Low physical activity level, %**	64.5	62.8	63.6	.75
**Fair or poor self-rated health, %**	61.7	40.3	51.1	.001
**Moderate or severe depressive symptoms,^c^ %**	27.0	28.0	27.5	.85
**Self-reported medical conditions, %**				
Depression/anxiety	55.6	49.0	52.3	.26
High cholesterol	62.7	67.0	64.8	.45
High blood pressure	73.2	67.6	70.4	.29
**Uninsured, %**	41.8	44.7	43.3	.63

Abbreviations: BMI, body mass index; SD, standard deviation.

a *P* value for χ^2^ test for categorical variables and *t* test for continuous variables.

b BMI >30 kg/m^2^.

c Patient Health Questionnaire depression scale (PHQ-8) score ≥10 (14).

We found no differences between the intervention and control groups in baseline blood pressure or lipid levels or in the number of outpatient clinic visits or emergency department encounters (Table 2). The control group was more likely to report a hospitalization during the prior year. The mean number of outpatient clinic visits in the past year was 9.2, and more than one-third reported an emergency department visit in the prior year.

**Table 2:** Changes in Primary and Secondary Outcomes for Participants From Baseline to 12 Months, Peer Support for Achieving Independence in Diabetes (Peer-AID), Seattle, Washington, 2010–2014^a^

Measure	Control (n = 142)			Intervention (n = 145)			Intervention Effect	
	Baseline	12 months	Difference (95% CI)	Baseline	12 months	Difference (95% CI)	Adjusted Difference (95% CI)	*P* Value
HbA_1c_, mean % (SD)	9.04 (1.92)	8.71 (2.15)	−0.33 (−0.68 to 0.03)	9.09 (1.66)	8.58 (1.88)	−0.51 (−0.88 to −0.15)	−0.14 (−0.58 to 0.30)	.54
Total cholesterol to HDL ratio^b^ (SD)	4.2 (1.8)	4.0 (1.1)	−0.2 (−0.5 to 0.2)	4.2 (1.1)	4.2 (1.3)	0.01 (−0.2 to 0.2)	0.2 (−0.4 to 0.8)	.53
Systolic BP, mean (SD), mm Hg	128.4 (20.1)	128.7 (19.5)	0.3 (−2.8 to 3.3)	131.0 (19.9)	130.3 (17.4)	−0.7 (−3.8 to 2.47)	−0.2 (−3.9 to 3.5)	.90
Diastolic BP, mean (SD), mm Hg	80.9 (11.5)	80.5 (11.0)	−0.5 (−2.6 to 1.5)	82.6 (10.5)	81.0 (9.2)	−1.6 (−3.5 to 0.3)	−0.2 (−2.5 to 2.2)	.85
BMI, mean (SD), kg/m^2^	35.0 (9.4)	34.6 (9.3)	−0.4 (−0.9 to 0.1)	32.4 (7.7)	32.6 (7.6)	0.3 (−0.3 to 0.9)	0.53 (−0.2 to 1.3)	.17
HRQOL, MCS score, mean (SD)	46.1 (12.1)	46.9 (14.2)	0.8 (−1.6 to 3.3)	48.1 (11.9)	49.9 (11.3)	1.9 (−0.4 to 4.2)	1.7 (−1.2 to 4.7)	.25
HRQOL, PCS score, mean (SD)	40.2 (10.6)	40.4 (11.5)	0.3 (−1.4 to 1.9)	39.7 (11.9)	42.1 (12.1)	2.4 (0.8 to 4.1)	1.9 (−0.2 to 4.1)	.07
Diabetes-specific HRQOL score, mean (SD)	3.0 (2.2)	3.1 (2.1)	0.04 (−0.3 to 0.4)	3.1 (2.3)	2.9 (2.1)	−0.3 (−0.7 to 0.1)	−0.3 (−0.7 to 0.2)	.22
Social burden subscale of Diabetes-39 score,^c^ mean (SD)	21.7 (23.3)	23.0 (25.9)	1.4 (−2.9 to 5.7)	21.2 (23.4)	17.4 (22.3)	−3.7 (−8.2 to 0.7)	−5.3 (−10.7 to 0.03)	.05
Outpatient clinic visits in past year, n (SD)	10.0 (11.3)	9.4 (10.2)	—	8.6 (8.8)	7.8 (6.3)	—	0.85 (0.79 to 0.93)^d^	<.001
ED visits in past year, n (SD)	0.6 (1.2)	0.7 (1.3)	—	0.8 (1.7)	0.7 (1.3)	—	0.83 (0.60 to 1.15)^d^	.26
Hospitalizations in past year, n (SD)	0.7 (2.3)	0.5 (0.9)	—	0.2 (0.6)	0.4 (0.8)	—	1.02 (0.65 to 1.59)	.95
Hospitalized in past year, n (%)	49 (35)	38 (28)	—	25 (17)	28 (22)	—	0.95^e^ (0.52–1.74)	.86
ED visit in past year, n (%)	46 (32)	46 (35)	—	59 (41)	49 (38)	—	1.12^e^ (0.64–1.96)	.69

Abbreviations: —, not applicable; BMI, body mass index; BP, blood pressure; CI, confidence interval; ED, emergency department; HbA_1c_, glycated hemoglobin A_1c_; HDL, high-density lipoprotein cholesterol; HRQOL, health-related quality of life; MCS, 12-item Short-Form Health Survey, Mental Component Summary scale (10); PCS, 12-item Short-Form Health Survey, Physical Component Summary scale (10); SD, standard deviation.

a Each outcome uses the analysis data set with observed baseline and 12-month values — “complete case.” Baseline and 12 months values are unadjusted.

b n = 252 (n = 123 in the control group and n = 129 in the intervention group). Data were missing for 35 participants.

c Diabetes-39 scales (11).

d Poisson zero-inflated model: multiplicative effect — mean number for the intervention group is *x* times the mean number for the control group.

e Odds ratio.

**Figure 2:**
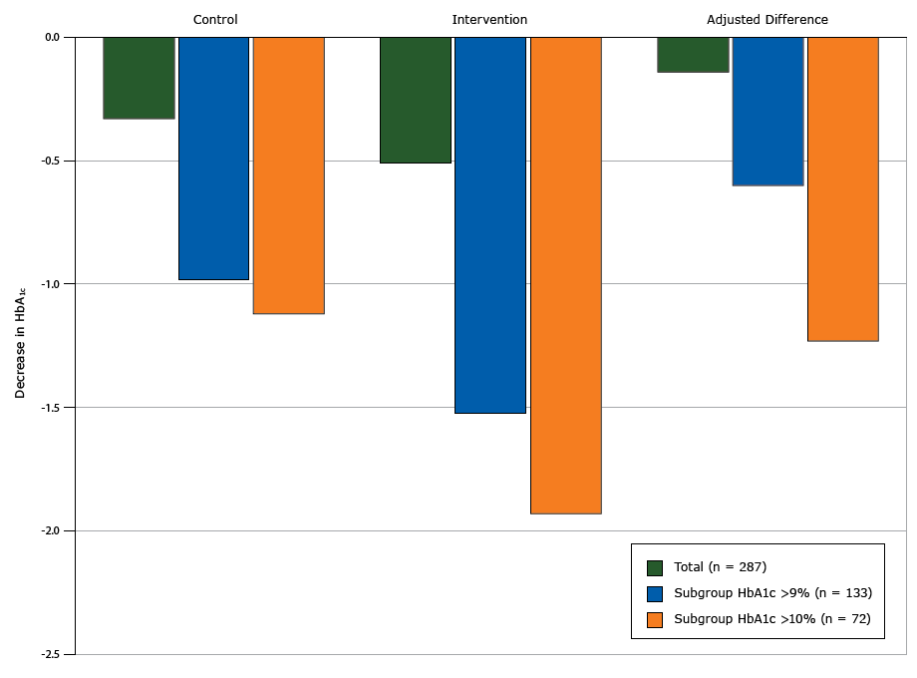
Decreases in glycated hemoglobin A_1c_ (HbA_1c_) from baseline to 12 months by intervention arm, total study population, subgroup with HbA_1c_ higher than 9%, and subgroup with HbA_1c_ higher than 10%, Peer Support for Achieving Independence in Diabetes (Peer-AID) trial using community health workers to provide self-management support among low-income adults with diabetes, Seattle, Washington, 2010–2014. *P* = .046 for the adjusted difference in HbA_1c_ value between the control and intervention groups for the subgroup with HbA_1c_ higher than 10%. **Table:** 

Table 2 reports the treatment effect for all primary and secondary outcomes. We found no change in HbA_1c_ values in the intervention group (from unadjusted mean of 9.09% to 8.58%, change of −0.51 points in HbA_1c_) compared with the control group (from unadjusted mean of 9.04% to 8.71%, change of −0.33 points) (*P* = .54). However we found a significant interaction between the baseline HbA_1c_ value and intervention group (*P* = .04), with an increasing treatment effect seen in people with higher HbA_1c_ values. In the subgroup analyses of individuals with a baseline HbA_1c_ value higher than 9%, the intervention group had a nonsignificant 0.60-point greater decrease in HbA_1c_ compared with the control group (Figure 2). In the subgroup analysis of individuals with a baseline HbA_1c_ value higher than 10%, the intervention group had a significant 1.23-point greater decrease in HbA_1c_ (*P* = .046) compared with the control group (Figure 2).

Population	Decrease in HbA_1c_		
	Control	Intervention	Adjusted Difference
**Total (n = 287)**	**−0.33**	**−0.51**	**−0.14**
Subgroup HbA_1c_ >9% (n = 133)	**−**0.98	**−**1.52	**−**0.60
Subgroup HbA_1c_ >10% (n = 72)	**−**1.12	**−**1.93	**−**1.23

Although some secondary outcomes (such as systolic and diastolic blood pressures, number of emergency department visits) improved more among intervention participants than among the control group, the differences were not significant (Table 2). We found a decrease in self-reported physician visits (15% lower in the intervention group, *P* < .001), no improvement in quality of life in the intervention group (increase in PCS scale of 0.25 in controls vs 2.4 in intervention group, *P* = .07), and a nonsignificant difference in the MCS scale. We found a decrease in reported social burden subscale of the Diabetes-39 instrument (*P* = .05) in the intervention group relative to the control group. We found no differences in other diabetes-specific quality-of-life scales.

## Discussion

In this randomized clinical trial among 287 low-income adults in a home-based CHW intervention to support diabetes self-management, HbA_1c_ did not significantly improve in the intervention group relative to usual care. Among participants with a baseline HbA_1c_ value higher than 10%, the mean decrease in HbA_1c_ in the intervention group was greater than in the control group. Intervention participants also reported significantly fewer outpatient clinic visits during the 12-month intervention period and a nonsignificant increase in HRQOL.

A recent meta-analysis reported modest benefit in glycemic control for CHW interventions of 12 months or longer among people with diabetes, with better results for people with poorer glycemic control at baseline (3). Other shorter-term CHW studies with 6 months of follow-up data showed a greater effect on HbA_1c_ values (21,22), as have studies that relied on medical record data alone to determine glycemic control (20). In our study we obtained both medical record and enrollment HbA_1c_ data and found that more than one-quarter of patients with elevated medical record HbA_1c_ values had significant improvement before enrollment, suggesting that calculating a preintervention/postintervention change in HbA_1c_ values based on medical records may overestimate intervention effects relative to using a baseline value for the preintervention value.

Although our study was not powered to detect a difference in HRQOL, we found a nonsignificant increase (of approximately 2 points) in the SF-12 PCS scale among all intervention participants. We also noted a nonsignificant difference in the MCS scale and in the social burden subscale of the Diabetes-39 instrument. Despite the importance of HRQOL (23), few other CHW studies have reported on this patient-reported outcome (24). Confirming the quality-of-life benefit would require an adequately powered study.

The strengths of our study include the enrollment of a diverse population of low-income individuals from 3 health systems. Peer-AID was conducted in participants’ homes, including all enrollment and blood-draw procedures. We used a home mail-in kit for blood glucose testing, a system that could be used by other outreach programs. The intervention was implemented in a “real world” public health department setting, suggesting replication in practice is feasible, with high retention rates and protocol compliance.

Our study has several limitations.. The study may have been underpowered, as the treatment effect size used in our power calculations was based on larger intervention effects reported in studies published when we designed our trial (25). We also did not note any effects on secondary outcomes. The small number of participants (n = 78) in the stratum with HbA_1c_ greater than 10% at baseline limited the power of the study to detect changes in secondary outcomes in this group. In addition, the mean blood pressures of participants were fairly well controlled at baseline. The high rate of outpatient visits in the study population may have reduced the effect of CHW visits, since patients were being seen frequently in a clinic. Several markers of health status (eg, fair or poor health ratings, likelihood of reporting a hospitalization in the past year) were worse in the control group, which may have biased the results to the null. The study employed only 2 CHWs, potentially limiting generalizability given variation in the attributes and skills among CHWs. We do not have administrative data to determine the accuracy of self-reported health care use. However, our results regarding lower outpatient utilization are consistent with previous studies that used administrative data to determine health care use (26). Finally, self-reported data were used for all behavioral measures, possibly resulting in socially desirable responses.

Several changes to the CHW intervention might enhance its effectiveness. Although the CHWs were connected to each clinic site, they were not integrated into clinical care teams. Better coordination of primary care and CHW self-management support activities could generate mutual reinforcement (27). We designed our study to test a low-intensity intervention (4 visits and 1 optional visit), which may have not have been potent enough to change disease self-management. Other positive trials have used many more visits (28); however, the number of visits in previous trials varied and the optimal number of contacts is unknown (3). Increasing the number of CHW contacts and adding interval telephone, digital prompts, or email contact might prove useful (29).

We found that it is feasible to deliver a CHW intervention using the health department as a hub to service multiple safety-net providers. This approach to providing in-home diabetes self-management support through CHWs improved glycemic control for people with severe hyperglycemia, even among patients who frequently participated in primary care. Next steps would include increasing intensity or enhancing care coordination to improve the effectiveness of the intervention in those with less severe hyperglycemia.
